# The Digestive Tract Injuries Caused by Acute Pesticide Poisoning From 2014 to 2024: A Mini Literature Review

**DOI:** 10.1155/emmi/9110457

**Published:** 2025-12-02

**Authors:** Jingjing Han, Yujie Guo, Yingying Xu, Boru Sun

**Affiliations:** Emergency Department, Shengjing Hospital of China Medical University, Liaoning, China

**Keywords:** digestive tract injuries, gastrointestinal bleeding, organophosphorus poisoning, pesticide poisoning

## Abstract

Acute pesticide poisoning is a significant public health issue, particularly in low- and middle-income countries where pesticides are commonly used in agriculture. While the neurological and cardiovascular effects of pesticide poisoning have been extensively studied, gastrointestinal injuries remain underexplored despite their severe complications, including bleeding, perforation, and obstruction. This mini-review examines the prevalence, mechanisms, and treatment of gastrointestinal injuries caused by acute pesticide poisoning from 2014 to 2024. Based on an analysis of 11 studies encompassing 38 cases across China, India, and Japan, we identified organophosphate compounds as the most common culprits of gastrointestinal damage. Mechanisms of injury involve multifactorial mechanisms, including the direct toxicity of pesticides, adverse therapeutic interventions such as atropine and gastric lavage, and systemic effects like endothelial dysfunction and hypoxia. Preventive strategies are discussed, including the use of proton pump inhibitors, careful atropine dosing, and balloon jejunal catheter placement to minimize complications. This review underscores the urgent need for further research to develop targeted preventive and therapeutic measures for gastrointestinal injuries caused by pesticide poisoning, aiming to improve patient survival and quality of life.

## 1. Introduction

The application of pesticides is widespread in agricultural production, making the prevention and management of pesticide poisoning particularly challenging. Globally, 14%–20% of suicides are attributed to pesticide poisoning, a phenomenon especially prevalent in low- and middle-income countries [1]. Despite a decline in suicide rates over the past two decades, China still accounts for a substantial number of suicides compared to other regions worldwide [2, 3]. Pesticide poisoning is the leading method of suicide in China, responsible for nearly half of all suicide-related deaths [4]. Similarly, high-income countries such as Japan [5] and South Korea [6] also face significant issues with pesticide-related suicides. Common pesticides implicated in poisoning and suicides include paraquat, diquat, dichlorvos, and glyphosate, with organophosphates being the most prevalent. Notably, gastrointestinal (GI) bleeding is more frequently observed in patients with organophosphate poisoning compared to those poisoned by other pesticides.

The persistent absorption of pesticides in the stomach can lead to varying degrees of GI symptoms, including nausea, vomiting, GI bleeding, or mucosal injury. Severe cases may result in hemorrhagic shock and death [7]. A report indicates that upper GI bleeding can occur as early as 30 min after poisoning, with some patients experiencing recurrent or multiple episodes of bleeding [8]. In the acute phase, mucosal damage to the esophagus, stomach, and duodenum occurs in nearly 100% of patients with organophosphate poisoning, often presenting as erosive lesions. Additionally, ulcer-related repetitive damage to the gastric mucosa and subsequent fibrotic proliferation from scar contraction can lead to pyloric obstruction, which may require gastric resection or reconstruction of the gastric outflow tract in some cases, severely affecting quality of life. Although GI bleeding is not a common symptom of pesticide poisoning, some reports suggest that it can be severe and even life-threatening [9, 10].

While numerous studies have focused on the neurological and cardiovascular effects of pesticide poisoning, research on GI damage remains relatively scarce. However, GI injuries can lead to severe complications such as GI bleeding, perforation, or stenosis. Currently, there is limited comprehensive understanding of the variability, mechanisms, and treatment options to GI symptoms caused by pesticide poisoning, with most available data derived from case reports. This study aims to review cases and research on pesticide-induced GI injury, providing insights to improve treatment for affected patients.

## 2. Materials and Methods

### 2.1. Eligibility Criteria

We included original research on pesticide-induced GI injury, covering case reports, epidemiological analyses, survival outcomes, and effects on both patients and responders. Eligible study types included case reports, cross-sectional studies, cohort studies, and letters to the editor. Additionally, journal articles, dissertations, conference abstracts, and proceedings were considered for inclusion. Studies were excluded if published in languages other than English, involved simulated case reports, lacked accessible full abstracts, reported on animal cases, or failed to provide information relevant to GI injuries.

### 2.2. Search Strategy

We searched the literature published from January 2014 to September 2024 in four databases: PubMed, Embase, ProQuest, and Web of Science. We used a combination of subject terms and free terms to search, with keywords including pesticide, herbicide, paraquat, diquat, dichlorvos, organophosphate poisoning, celphos, aluminum phosphide, GI lesions, GI hemorrhage, peptic ulcer, gastric ulcer, esophageal ulcer, lower intestinal bleeding, etc. The complete search strategy is shown in Supplementary 1.

### 2.3. Study Selection and Data Extraction

Following the removal of duplicate records, a two-phase screening process was conducted to identify studies meeting the inclusion criteria. Initially, two independent reviewers assessed the titles and abstracts based on predefined eligibility criteria. In the subsequent phase, the full texts of studies deemed potentially relevant were independently evaluated. Discrepancies between reviewers were resolved through discussion and consensus to ensure accuracy and rigor in the selection process.

A structured data extraction form was developed to systematically capture relevant information from the included studies. For case reports, the form encompassed fields such as author and publication year, country, patient demographics, pesticide name, concentration and dosage, poisoning-related treatments, digestive tract injury types and manifestations, digestive tract injury-related tests, examinations, treatments, and patient outcomes. Data extraction was performed by one reviewer using this form, with a second reviewer independently verifying the extracted data to ensure accuracy and reliability. For noncase report studies, the same extraction framework was adapted to record exposure type, outcome measures, and key findings relevant to GI injury.

## 3. Results

**Figure 1:**
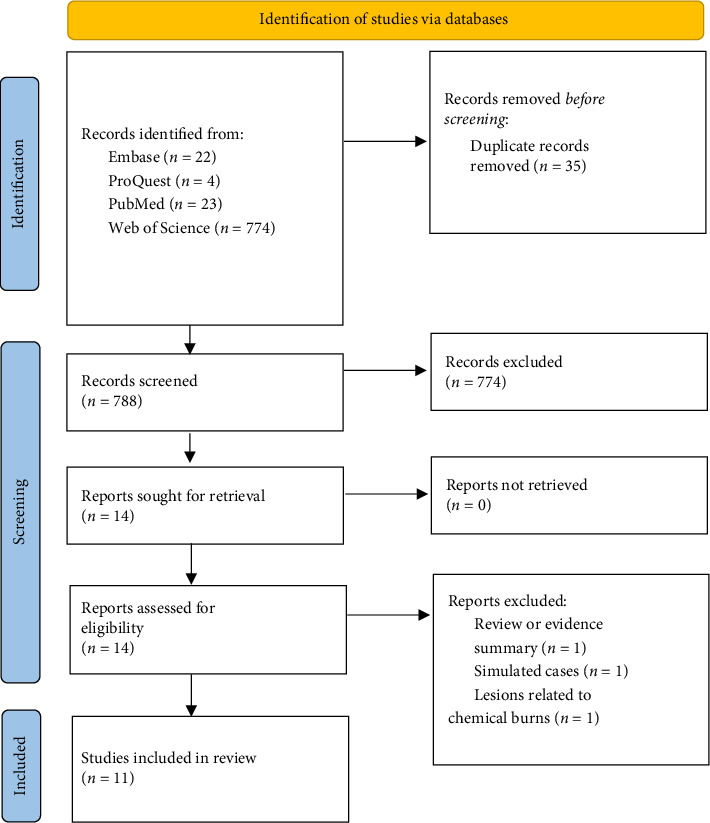
PRISMA flow diagram of study selection process.

A total of 823 citations were retrieved from electronic databases. After removing duplicates using EndNote X9, 775 unique citations remained, of which 14 studies were selected for full-text screening and 11 were ultimately included (Figure 1) [7, 10–19]. These included nine journal articles and two conference abstracts or proceedings. The included studies, spanning the past decade, reported on 38 cases from 11 reports. These studies originated from three countries: China, India, and Japan, with India contributing the largest number of publications.

Among the cases with reported gender, eight patients were male and five were female, with a median age of 32 years (interquartile range: 28–38 years). As shown in Table 1, the most frequently implicated pesticide was glyphosate herbicide, which was involved in six cases across multiple studies [7, 16, 19]. Dichlorvos, an organophosphate pesticide, was reported in three studies [10, 14, 18]. Paraquat exposure was described in two studies [13, 17], and aluminum phosphide was identified in three studies [11, 12, 15].

The types of GI injuries varied in severity and type. GI bleeding was the most common manifestation, occurring in nine cases [7, 11, 14, 15, 19] and often presenting as hematemesis, melena, or significant hemorrhagic shock. Gastric or esophageal ulcers were observed in five cases [16, 19], some of which progressed to pyloric obstruction or stricture formation. Pyloric obstruction and cardia-pylorus stenosis, characterized by stiffness and structural narrowing of the gastric outlet, were reported in three cases [19]. Intestine perforations were identified in two cases [17], both requiring emergency surgical intervention. Other reported injuries included oropharyngeal erosion following paraquat ingestion [16], intestinal gangrene associated with aluminum phosphide poisoning [12], and esophageal injury classified using the corpus esophageal injury grading system, described in a study involving 25 patients [13].

Management strategies varied based on the severity of injury. Gastric lavage and hemoperfusion were commonly used initial interventions for pesticide poisoning but were associated with additional GI complications. Surgical interventions were necessary in multiple cases, including subtotal gastrectomy, bowel resection, and digestive tract reconstruction. In cases of GI bleeding, blood transfusion was frequently administered. Despite treatment efforts, mortality was high (6/13), particularly among patients with hemorrhagic shock or severe perforations. Fatal outcomes were reported in cases involving aluminum phosphide [11, 12, 15]. Dichlorvos [10, 14, 18], and severe hemorrhagic complications from glyphosate poisoning [16]. Patients who survived were often discharged with complications such as long-term drainage tubes or required additional surgical interventions.

## 4. Discussion

GI injury caused by acute pesticide poisoning may manifest in various forms, including esophageal strictures, upper GI bleeding, small intestinal perforation, pyloric obstruction, and intestinal obstruction. In cases of acute small intestinal perforation, patients may succumb to the condition within a few days [10]. Therefore, understanding the etiology, clinical manifestations, and preventive and therapeutic measures of GI injury is critically important.

### 4.1. Mechanisms of Digestive Tract Injury Induced by Acute Pesticide Poisoning

The mechanism of GI damage caused by acute pesticide poisoning is the result of multifactorial mechanisms, encompassing the direct toxicity of the chemical agents, adverse effects of therapeutic interventions, and systemic pathological changes. Alkaline compounds cause more severe damage to the digestive system compared to acidic compounds. Alkaline corrosive agents can induce protein dissolution and fat saponification, resulting in liquefactive necrosis, which allows deeper tissue penetration and extensive damage. Additionally, alkali absorption leads to thrombosis in blood vessels, impeding blood flow to already damaged tissue [20]. Acute exposure to alkali can cause marked mucosal cellular and stromal edema in the esophagus of mice [21]. In addition, due to the relatively high surface tension of alkaline liquids, they tend to remain in contact with tissues for a longer duration, thereby exacerbating the extent of injury [22]. In contrast, strong acids primarily lead to coagulative necrosis by denaturing proteins and forming an eschar that can limit further tissue penetration [20]. The direct toxic effects on mucosa primarily manifest as ulcers and erosion of the oropharyngeal and GI mucosa. Examples include the release of phosphine gas leading to localized inflammation, esophageal strictures [11, 18, 23], and widespread GI bleeding and ulceration induced by alkaline phosphatase [24], commonly observed in aluminum phosphide poisoning cases. Additionally, the retention of tablets within the esophagus exacerbates localized corrosion, usually caused by exposed tablets [25]; even intact tablets may contribute to esophageal damage, suggesting that components other than phosphine gas may also play a significant role in causing injury [24]. Toxic-induced exfoliative esophagitis is a distinct type of esophageal injury. The underlying mechanism is generally attributed to the relatively loose structure of the esophageal mucosa and submucosa, where corrosive necrosis caused by toxic substances leads to the detachment of the superficial mucosal layer (stratified squamous epithelium) from the lamina propria. Triggers such as severe vomiting can exacerbate the condition, resulting in complete separation of the entire superficial mucosal layer from the underlying lamina propria [26].

In therapeutic interventions, gastric lavage—especially when repeated frequently or performed over an extended period—can lead to excessive gastric distension, overstretching of the gastric wall, and mechanical damage caused by the lavage apparatus [27]. During the treatment of pulmonary edema and shock, the administration of high doses of dexamethasone may harm the gastric mucosa, potentially resulting in stress ulcers. Additionally, atropine treatment can lead to paralytic ileus. When atropine is administered, the toxin may have already reached the small intestine, and the atropine-induced reduction in intestinal motility prolongs the contact time between the toxin and the small intestinal mucosa, exacerbating the extent of injury. Mechanistically, atropine acts as a muscarinic receptor antagonist, particularly inhibiting M3 receptors on intestinal smooth muscle cells. This inhibition suppresses peristalsis and intestinal secretions, delaying the clearance of ingested toxins. The extended toxin-mucosa interaction increases the risk of mucosal irritation, ischemia, and subsequent ulceration or necrosis [28].

Moreover, organophosphate poisoning frequently induces endothelial dysfunction, leading to microvascular thrombosis, ischemic necrosis of the intestinal wall, and delayed ischemic lesions. In severe cases, these complications may progress to intestinal perforation, segmental gangrene, and ultimately fecal peritonitis and sepsis [9, 10, 12]. In cases of severe poisoning, hemodynamic alterations and hypoxia further exacerbate ischemic tissue damage. Abdominal organs, such as the pancreas, may also sustain injury, potentially resulting in the formation of pseudocysts that compress the intestines and other organs, worsening the clinical condition [18]. These intricate mechanisms underline the extensive and severe nature of GI damage caused by acute pesticide poisoning, necessitating early multidimensional assessment and integrated therapeutic approaches to mitigate fatal outcomes.

### 4.2. Prevention of Digestive Tract Injuries in Acute Pesticide Poisoning

The treatment of GI injuries caused by acute pesticide poisoning depends on the severity of the damage and may involve surgical intervention or conservative measures such as blood transfusion. However, research on the prevention of severe GI injuries in acute pesticide poisoning remains relatively limited, despite the significant threat these injuries pose to both patient survival and postrecovery quality of life. Future studies could focus on strategies to prevent or mitigate GI damage caused or exacerbated by pesticides or treatment interventions during detoxification.

Research indicates a positive correlation between the APACHE II score and upper GI bleeding [8]. Special attention should be given to patients with low APACHE II scores to monitor for symptoms of upper GI bleeding. Prophylactic administration of proton pump inhibitors (PPIs), such as omeprazole (40 mg daily), significantly reduces the incidence of stress ulcer bleeding and improves patient outcomes [29]. Platelet aggregation is impaired in an acidic environment, and when the gastric pH drops below 6, aggregation is almost completely abolished [30]. PPIs have been shown to maintain intragastric pH above 6 for 84%–99% of the time within 24 h [31]. Meta-analyses have demonstrated that, compared with placebo or H2-receptor antagonists, intravenous PPIs significantly reduce the recurrence of ulcer rebleeding and the need for surgical intervention [32]. Although randomized controlled trial evidence on the prophylactic use of PPIs specifically in organophosphate poisoning is limited, one Chinese study showed that pantoprazole significantly lowered the incidence of upper GI bleeding in patients with severe acute organophosphate pesticide poisoning (pantoprazole: 7/58 vs. famotidine: 23/51) [33]. Additionally, in a meta-analysis of patients with gastroduodenal ulcers who received endoscopic hemostasis, those treated with intravenous pantoprazole had significantly lower rebleeding rates (3.2%–6.7%) compared to those receiving intravenous ranitidine (12.9%–16.0%; *p* < 0.05) [34].

The key to treating upper GI bleeding in acute organophosphate poisoning lies in the rational use of atropine. Dosage adjustment based on poisoning severity is crucial: achieving atropinization within 4–6 h for mild cases, 3–5 h for moderate cases, and 1–2 h for severe cases. This approach prevents atropine toxicity and exacerbated bleeding in mild cases while ensuring rapid atropinization in severe cases [35]. The duration of atropinization should not be prolonged unnecessarily; once toxins are cleared, the atropine dosage should be gradually reduced to avoid persistent bleeding. As for nonpharmacological interventions, the use of a balloon jejunal catheter offers an additional preventive approach. This device involves a gastric decompression tube located within the stomach and a jejunal catheter positioned distal to the ligament of Treitz. This dual system enables simultaneous gastric decompression and enteral nutritional support, which enhances nutritional status while minimizing complications such as GI bleeding, perforation, esophageal strictures, and esophagotracheal fistulas [36]. However, it is important to note that current evidence regarding the use of balloon jejunal catheters is limited to a small number of cases from a single center in China. As such, the feasibility, safety profile, and generalizability of this approach remain uncertain. Potential risks, such as catheter displacement, infection, or tube-related complications, warrant careful consideration. Larger, multicenter studies are needed to validate its efficacy and safety before widespread clinical adoption.

### 4.3. Limitations

One of the primary limitations of this review is the small sample size, with only 38 cases included from 11 studies spanning China, India, and Japan. This limited sample size may affect the generalizability of our findings, as it does not fully capture the spectrum of GI injuries caused by pesticide poisoning across different populations and healthcare settings. The small number of cases may also introduce selection bias, as studies with severe or unusual presentations may be more likely to be reported. Furthermore, the heterogeneity in pesticide types, exposure doses, and treatment protocols across studies may limit the ability to draw definitive conclusions regarding the mechanisms and management strategies for pesticide-induced GI injuries. Future large-scale, multicenter studies and systematic registries are needed to provide more comprehensive data on the prevalence, risk factors, and optimal treatment approaches for GI injuries in pesticide poisoning. Additionally, prospective cohort studies could help establish causal relationships between pesticide exposure, treatment interventions, and GI outcomes, ultimately leading to improved clinical management strategies. Another important limitation of this review is the potential geographical bias, as a majority of the included studies originate from India. This overrepresentation may influence the findings due to regional variations in pesticide usage patterns, healthcare infrastructure, treatment protocols, and reporting standards. Differences in healthcare accessibility, emergency response capabilities, and diagnostic practices may affect the detection, management, and reporting of GI injuries following pesticide poisoning. Future research should aim for a more diverse representation of cases from various geographic regions to better understand global patterns of pesticide-induced GI injuries. Multicenter international collaborations and standardized reporting criteria could help mitigate this bias and provide a more comprehensive understanding of the problem.

## 5. Conclusions and Future Directions

This review highlights the significant GI injuries caused by acute pesticide poisoning, a serious public health issue, and particularly in low- and middle-income countries. Although GI complications are less studied compared to neurological and cardiovascular effects, they can lead to severe outcomes, including bleeding, perforation, and obstruction. Preventive measures, including judicious atropine use, PPI administration, and balloon jejunal catheter placement, have been proposed to mitigate GI damage. However, current evidence is limited and primarily based on case reports or small series.

To advance the field, further research should prioritize multicenter prospective studies to validate the efficacy of preventive intervention such as PPI and balloon catheters in acute pesticide poisoning. Additionally, mechanistic studies exploring novel therapeutic targets—such as specific antidotes for glyphosate or paraquat-induced GI injury—are warranted. Emphasis should also be placed on the integration of effective GI injury prevention protocols into existing toxicology treatment guidelines. Such translational efforts have the potential to standardize care, reduce complications, and ultimately improve patient survival and quality of life.

## Figures and Tables

**Table 1:** Characteristics of included digestive tract injuries caused by acute pesticide poisoning cases described in detail.

Author (Year)	Country	Patients		Pesticide poisoning			Digestive tract injury				Patients' outcome
		Age (years)	Sex	Type	Concentration and dosage	Treatments	Type	Manifestations	Tests and examinations	Treatments	
Ren et al., (2020a) [7]	China	36	Female	Glyphosate herbicide	Unknown	Hemoperfusion	GI bleeding	Abdominal pain and vomited 10 mL of blood gastric juice	—	—	Survive to discharge
		53	Female	Glyphosate herbicide	80 mL	Gastric lavage and blood perfusion	Gastric ulcer and pyloric obstruction	—	—	Bi II subtotal gastrectomy	Survive to discharge
		28	Male	Glyphosate herbicide	300 mL	Gastric lavage and blood perfusion	Stiffness of submucosal vascular texture, stricture of cardia, stiffness of whole stomach and cavity, and stricture of pyloric entrance	—	—	—	/

Ren et al., (2020b) [19]	China	36	Male	Glyphosate herbicide	120 mL oral glyphosate, 50 mL triazolone	Hemoperfusion	Extensive ulcers in the whole gastric mucosa; GI hemorrhage	Dark red blood was detected in the GI tract during the operation	Gastroscopy	Total gastrectomy and digestive tract reconstruction	Died of digestive tract hemorrhage and hemorrhagic shock
		31	Female	Glyphosate herbicide	240 mL	Gastric lavage; systematic treatment	GI bleeding	100–700 mL/day recurrent stool bleeding	—	Large blood transfusion	Died of hemorrhagic shock
Lu et al., (2021) [18]	India	33	Male	Dichlorvos	Unknown	Gastric lavage and atropine treatment	Duodenal perforation	Intensified abdominal pain after oral ingestion of a small amount of a fluid diet	Gastroscopy	Fasting and conservative treatment	Discharged with a drainage tube
Inaba et al., (2020) [17]	Japan	—	Female	Paraquat	10 mL	—	Erosion of the oral cavity and pharynx; linear ulcers of the esophagus and stomach	Oral erosion and hoarseness	Upper GI endoscopy	—	Survive to discharge
Hung et al., (2021) [16]	China	78	Female	Glyphosate isopropylammonium and benzodiazepine	Unknown	—	Delayed lower intestinal bleeding	Intermittent bloody stool	Upper GI endoscopy and colonoscopy; exploratory laparotomy	Resected 100 cm of the ileum till the healthy bowel and reconstructed it with side-to-side ileocecal anastomosis	Survive to discharge
Hugar et al., (2015) [15]	India	45	Male	Aluminum phosphide	Five tablets (3 g/each) with 56% aluminum phosphide	—	Stomach and small intestine bleeding			—	Expired
Gupta et al., (2018) [14]	India	14	Male	Dichlorvos	50 mL, 76% dichlorvos	—	Upper GI bleeding	Massive hematemesis and melaena	Upper GI endoscopy	Blood transfusion	Survive to discharge
Chandel et al., (2022) [12]	India	18	Male	Aluminum phosphide	10 g of 56% aluminum phosphide	Intravenous fluids and pantoprazole infusion	Bowel gangrene	Dark, red-colored bleeding per rectum along with gradual abdominal distension and pain in the abdomen	Digital rectal examination, proctoscopy; upper GI and lower GI endoscopy	Bowel resection	Expired
Behera et al., (2017) [11]	India	30	Male	Aluminum phosphide	One tablet	Intubated, gastric lavage with potassium permanganate and magnesium sulfate infusion	GI bleeding (second day)	Vomited about 500 mL of dark colored blood	—	Exploratory laparotomy, adhesiolysis, gastrostomy closure with feeding jejunostomy and drainage	Expired
Mahajan et al., (2016) [10]	India	28	Male	Dichlorvos	Unknown	Atropine, gastric lavage, decontamination with activated charcoal	Small intestine perforations (six perforations)	Progressively worsening abdominal distension, loose stools and high nasogastric aspirates	Abdomen CT, emergency exploratory laparotomy	The involved portion of the bowel was resected and reanastomosed	Succumbed to this illness
Chandel et al., (2021) [13]	India	Pesticide type: paraquat; digestive tract injury: esophageal injury 25 patients underwent upper GI endoscopy: 12 (48%) oral ulcers, 17 (68%) dysphagia, 18 (72%) vomiting, 6 (24%) hematemesis Corpus esophageal injuries grade: 2a in 12 (48%) cases, 2b in 7 (28%) cases, 3a in 6 (24%) cases									

Abbreviation: GI, gastrointestinal.

## Data Availability

Data sharing is not applicable to this article as no new data were created or analyzed in this study.

## Supporting Information

Supplementary 1: Detailed search strategies.
