# Supplementary material for: The Digestive Tract Injuries Caused by Acute Pesticide Poisoning From 2014 to 2024: A Mini Literature Review
Source: Emerg Med Int. 2025 Dec 2;2025:9110457. doi: 10.1155/emmi/9110457 (PMC12688633; doi:10.1155/emmi/9110457)
Supplement: Supporting Information — Additional supporting information can be found online in the Supporting Information section. [file 9110457.f1.docx]

**PubMed 20240929 - 23 results**

("pesticide"[Title/Abstract] OR "Pesticides"[Title/Abstract] OR "herbicide"[Title/Abstract] OR "Herbicides"[Title/Abstract] OR "Paraquat"[Title/Abstract] OR "Diquat"[Title/Abstract] OR "Dichlorvos"[Title/Abstract] OR "Organophosphate Poisoning"[Title/Abstract] OR "organophosphorus poisoning"[Title/Abstract] OR "celphos"[Title/Abstract] OR "aluminum phosphide"[Title/Abstract] OR ("Pesticides"[MeSH Terms] OR "Herbicides"[MeSH Terms] OR "Paraquat"[MeSH Terms] OR "Diquat"[MeSH Terms] OR "Dichlorvos"[MeSH Terms] OR "Organophosphate Poisoning"[MeSH Terms])) AND ("gastrointestinal lesions"[Title/Abstract] OR "digestive tract injury"[Title/Abstract] OR "Gastrointestinal Hemorrhage"[Title/Abstract] OR "gastrointestinal bleeding"[Title/Abstract] OR "Peptic Ulcer"[Title/Abstract] OR "gastric ulcer"[Title/Abstract] OR "Stomach Ulcer"[Title/Abstract] OR "esophageal ulcer"[Title/Abstract] OR "lower intestinal bleeding"[Title/Abstract] OR "intestine ulcer"[Title/Abstract] OR "bowel gangrene"[Title/Abstract] OR ("Gastrointestinal Hemorrhage"[MeSH Terms] OR "Peptic Ulcer"[MeSH Terms] OR "Stomach Ulcer"[MeSH Terms]))

**Embase 20240929 - 22 results**

#1

pesticide:ti,ab,kw OR pesticides:ti,ab,kw OR herbicide:ti,ab,kw OR herbicides:ti,ab,kw OR paraquat:ti,ab,kw OR diquat:ti,ab,kw OR dichlorvos:ti,ab,kw OR 'organophosphate poisoning':ti,ab,kw OR 'organophosphorus poisoning':ti,ab,kw OR 'celphos':ti,ab,kw OR 'aluminum phosphide':ti,ab,kw

#2

'gastrointestinal lesions':ti,ab,kw OR 'digestive tract injury':ti,ab,kw OR 'gastrointestinal hemorrhage':ti,ab,kw OR 'gastrointestinal bleeding':ti,ab,kw OR 'peptic ulcer':ti,ab,kw OR 'gastric ulcer':ti,ab,kw OR 'stomach ulcer':ti,ab,kw OR 'esophageal ulcer':ti,ab,kw OR 'lower intestinal bleeding':ti,ab,kw OR 'intestine ulcer':ti,ab,kw OR 'bowel gangrene':ti,ab,kw

#1 AND #2

**ProQuest 20240929 - 4 results**

summary("pesticide" OR "Pesticides" OR "herbicide" OR "Herbicides" OR "Paraquat" OR "Diquat" OR "Dichlorvos" OR "Organophosphate Poisoning" OR "organophosphorus poisoning" OR "celphos" OR "aluminum phosphide") AND summary("gastrointestinal lesions" OR "digestive tract injury" OR "Gastrointestinal Hemorrhage" OR "gastrointestinal bleeding" OR "Peptic Ulcer" OR "gastric ulcer" OR "Stomach Ulcer" OR "esophageal ulcer" OR "lower intestinal bleeding" OR "intestine ulcer" OR "bowel gangrene")

**Web of Science 20240929 - 774 results**

(TS=("pesticide" OR "Pesticides" OR "herbicide" OR "Herbicides" OR "Paraquat" OR "Diquat" OR "Dichlorvos" OR "Organophosphate Poisoning" OR "organophosphorus poisoning" OR "celphos" OR "aluminum phosphide")) AND TS=("gastrointestinal lesions" OR "digestive tract injury" OR "Gastrointestinal Hemorrhage" OR "gastrointestinal bleeding" OR "Peptic Ulcer" OR "gastric ulcer" OR "Stomach Ulcer" OR "esophageal ulcer" OR "lower intestinal bleeding" OR "intestine ulcer" OR "bowel gangrene")
